# Evaluation of Single-Shade Composite Resin Color Matching on Extracted Human Teeth

**DOI:** 10.1155/2023/4376545

**Published:** 2023-06-26

**Authors:** Erika Thaís Cruz da Silva, Caroline de Farias Charamba Leal, Samille Biasi Miranda, Mariana Evangelista Santos, Sônia Saeger Meireles, Ana Karina Maciel de Andrade, Marcos Antonio Japiassú Resende Montes

**Affiliations:** ^1^Department of Restorative Dentistry, School of Dentistry, University of Pernambuco (UPE), Recife, Brazil; ^2^Departmentof Operative Dentistry, Federal University of Paraíba (UFPB), João Pessoa, Paraíba, Brazil

## Abstract

**Introduction:**

Universal single-shade composite resins are characterized by a property that enables the creation of restorations that mimic tooth structure to the extent possible with fewer shades of color.

**Objectives:**

This study aimed to instrumentally and visually evaluate the color correspondence of two single-shade composite resins in extracted human teeth multishade composite resins.

**Methods:**

Upper central incisors and upper and/or lower molars with intact buccal surfaces were selected. The study consisted of a control group (*n* = 20): Z250 XT (3M ESPE) (G1) multishade composite resin in colors A1 to A4, and a test group (*n* = 20) divided further into two equal groups, consisting of single-shade composite resin Omnichroma (Tokuyama Dental) (G2) and single-shade composite resin Vittra APS Unique from (FGM) (G3). Instrumental evaluation was performed using a spectrophotometer, and visual evaluation was performed by three observers. Descriptive measurements related to the differences in color obtained through instrumental means were analyzed using mean and standard deviation, wherein the means were compared using ANOVA, applying the Bonferroni post hoc test.

**Results:**

A statistically significant difference was observed among the groups (G1, G2, and G3) (ANOVA: *p* < 0.001). For the visual assessment, regardless of the assessment group, 77.49% of the teeth were within the acceptable color-match classification, with the single-shade resins showing better correspondence than the multishade resins.

**Conclusion:**

Single-shade composite resins showed different color-matching results when compared to multishade resins, both in spectrophotometry and visual evaluations. *Clinical Significance*. Single-shade composite resins simplify the shade-selection process and are promising materials for use in dental practice.

## 1. Introduction

For a restoration to be esthetically acceptable, the color of the composite resin and natural tooth structure must be so similar that the human eye cannot detect the difference between the two [1]. Layering techniques have been recommended for composite resin restorations. Although this technique is a very effective method for color matching, it requires considerable professional skill and more clinical time to perform [2]. Therefore, the emergence of new techniques is expected to facilitate clinical protocols, reduce clinical time, and facilitate the color selection process in dentistry, which is challenging.

Based on this color combination concept, single-shade composite resins have recently been introduced to the market. These materials are produced to perfectly match the surrounding tooth color, regardless of the color of the tooth to be restored [3]. These resins exhibit a phenomenon called the “chameleon effect” or “mixing effect,” which refers to the ability of a material to combine and acquire a color similar to that of its surrounding structures [4]. This means that two colors, when seen side by side, will mix under the right conditions so that the perceived color of a region changes to that of the surrounding area [5]. These composite resins have the advantage of being able to simulate all shades of tooth color using only a single shade [4]. All the aforementioned characteristics make these materials very promising for use in clinical practice, particularly when compared to materials that require multiple shades to accomplish extensive restorations [6].

Omnichroma (OC), single-color composite resin, was the first genuinely developed single-tone composite resin purported to have the potential to match all 16 Vita Classical shades, ranging from A1 to D4 [2]. OC resin was developed with smart color technology and uses the structural color concept, wherein the material itself weakens or amplifies specific wavelengths of light to blend with tooth color, unlike other composite resin systems that add red and yellow pigments to color the material [7].

Some studies have already evaluated the in vitro color matching or color-setting potential of these single-shade resins and obtained positive results (acceptable color matching) by making composite resin specimens. References [8–10] for dentures and artificial teeth [5, 11, 12]. Other in vitro studies have obtained negative results (unacceptable color matching) for acrylic denture teeth [2, 7]. In a clinical study, Nagi and Moharam [13] evaluated the color matching of OC single-tone resin in patients with class V and/or class III carious lesions and obtained a negative result, that is, an unacceptable color matching. Kobayashi et al. [14] performed a study with extracted human incisor teeth and reported that single-tone composite resins showed excellent color adaptation. Altınışık and Özyurt [4] evaluated the visual (CAP-V) and instrumental (CAP-I) color-matching potential in forty human incisors extracted from four single-tone composite resins (OC, Charisma Diamond One, Vittra Unique, and Essentia Universal) and obtained positive color-setting potential results for all of those tested.

However, no studies have evaluated the color matching of these single-shade resins by comparing them to a multishade composite resin in extracted human teeth. The aim of the present study is to instrumentally and visually evaluate the color correspondence of two single-shade composite resins in extracted human teeth, compared to a multishade composite resin. The null hypothesis is that there is no difference in color matching between of teeth restored with the single-shade and the multishade composite resins used in the study.

## 2. Materials and Methods

This study was approved (CAAE: 59543422.7.0000.5188) by the Ethics and Research Committee of the Federal University of Paraiba (UFPB). An in vitro study was performed to evaluate the color matching of OC (Tokuyama Dental, Tokyo, Japan) and Vittra APS Unique (FGM, Joinville, Brazil) single-tone composite resins in extracted human teeth. Forty teeth with different colors, upper central incisors, and upper and/or lower molars with intact buccal surfaces were selected. Teeth with carious lesions near the buccal surface and also teeth with major color changes were excluded from the sample. The number of specimens was defined based on previous studies [14], as recommended by the RoB assessment tool for laboratory studies on dental materials [15]. The study consisted of a control group (G1) (*n* = 20) restored with multishade composite resin Z250 XT (3M ESPE, St. Paul, MN) in shades A1, A2, A3, A3.5, and A4 in the corresponding colors and a test group (*n* = 20) split further into two equal groups (*n* = 10) for the OC (G2) and Vittra APS Unique single-shade composite resins (G3). The materials used in this study are listed in Table 1.

### 2.1. Restorative Procedure

A standardized cylindrical cavity (4 mm diameter and 2 mm depth) was made in the center of the buccal face of the teeth with cylindrical diamond bur number 1016 (KG Sorensen, Cotia, SP, Brazil) in a high-speed turbine under cooling. After making the cavities, the depth and diameter were measured using a millimeter probe and caliper, respectively. Following this, the surface was etched with 37% phosphoric acid (Condac37, FGM) for total acid etching for 15 s; then, a jet of water was applied for 15 s for washing before drying with jets of air. The universal adhesive system (Single Bond Universal, 3M ESPE) was applied with the aid of a microbrush applicator (KG Brush, FGM) using friction movement on the tooth structure for 20 s. Then, a brief jet of air was applied over the bonding agent for 5 s before the adhesive system was light cured for 10 s with the Valo Grand light-curing device (Ultradent, South Jordan, USA) at a light intensity of 1000 mW/cm^2^. The teeth were restored by inserting composite resin into the cavities in a single increment.

All composite resins were light cured using the Valo Grand light-curing device at a light intensity of 1000 mW/cm^2^ for 20 s. The light tip of the device was positioned perpendicular to the buccal surface of the tooth, and its diameter ensured adequate coverage and irradiation. A sequence of diamond discs with a diameter of 16 mm (Discos de Lixa, TDV, Pomerode/SC, Brazil) was used for finishing and polishing, carried out by the same operator under refrigeration, and in decreasing granulation for 10 s each. Specimens were stored in distilled water for 72 h before color measurements.

### 2.2. Instrumental Assessment

The tooth color was evaluated using a spectrophotometer (Easyshade Advance 4.0, VITA Zahnfabrick, Bad Säckingen, Germany) in the “individual tooth” function on a black background. The device was calibrated every three measurements. The CIELab *L*^*∗*^, *a*^*∗*^, and *b*^*∗*^ color coordinates defined by the International Commission on Illumination (CIE), which are widely used in dental literature, were obtained. The *L*^*∗*^ value determines the psychometric brightness from black to white (achromatic coordinate). The *a*^*∗*^ (green-red coordinate) and *b*^*∗*^ (blue-yellow coordinate) values are the psychometric chroma coordinates that indicate hue and chroma [16]. The measurements were repeated thrice for each specimen, and the data values were calculated. To obtain a better correlation with visual perception, the International Standard Organization (ISO) and CIE jointly recommended the use of the CIEDE 2000 color difference formula to calculate the total color difference, which is also based on the CIELAB color space [10]. Furthermore, recent studies concluded that the CIEDE 2000 formula reflects color differences perceived by the human eye better than CIELab [17–21]. Therefore, in this study, the difference in color was calculated using two parameters: CIEDE 2000 (ΔE 00) and CIELAB (ΔE ab).

The CIELAB color difference (ΔE ab) was evaluated by using the following equation:

(1) $$\begin{array}{c} {∆E}_{ab}=\sqrt{∆L^{*2}+∆a^{*2}+∆b^{*2}}\text{.} \end{array}$$

The CIEDE 2000 color difference (∆E 00) was calculated using an Excel spreadsheet implementation of the formula provided by Sharma [22].

(2) $$\begin{array}{c} {∆E}_{00}={\left[{\left(\frac{∆L^{\prime }}{K_{L}S_{L}}\right)}^{2}+{\left(\frac{∆C^{\prime }}{K_{C}S_{C}}\right)}^{2}+{\left(\frac{∆H^{\prime }}{K_{H}S_{H}}\right)}^{2}+RT\left(\frac{∆C^{\prime }}{K_{C}S_{C}}\right)\left(\frac{∆H^{\prime }}{K_{H}S_{H}}\right)\right]}^{1/2}\text{.} \end{array}$$

*ΔL′*, *ΔC′*, and *ΔH′* are the differences in lightness, chroma, and hue between the compared specimens, respectively. RT is a rotation function that explains the interaction between the chroma and hue differences in the blue region. *S*_*L*_, *S*_*C*_, and *S*_*H*_ are the weighting functions, whereas *K*_*L*_, *K*_*C*_, and *K*_*H*_ are the correct terms to be adjusted according to the experimental conditions [16]. The parametric factors *K*_*L*_, *K*_*C*_, and *K*_*H*_ are the correction terms for variation under experimental conditions and are all set at 1.0, under reference conditions determined by the CIE technical report.

Color adaptability was determined from the color differences between the teeth and composites across the thresholds of 50% : 50% perceptibility (PT) and 50% : 50% acceptability (AT), according to ISO/TR 28642:2016. The 50% : 50% color difference PT threshold represents the difference in color that can be detected by 50% of observers under controlled conditions, with the other 50% of observers noticing no difference in color between the compared objects. The 50% : 50% color difference AT threshold represents the color difference that is considered acceptable by 50% of observers under controlled conditions, with the other 50% of observers replacing or correcting the restoration.

The ΔEab values were evaluated in terms of 50% : 50% PT and 50% : 50% AT, and the thresholds were set at 1.2 and 2.7, respectively. The ΔE 00 values were also evaluated in terms of 50% : 50% PT and 50% : 50% AT, and the thresholds were set at 0.8 and 1.8, respectively, as reported in previous studies [23–25].

### 2.3. Visual Assessment

Visual assessments were performed by three female observers. Women are more sensitive to color differences than men [26]. The three evaluators selected for the study had experience and superior competence in esthetic restorative dentistry. All observers were previously calibrated and demonstrated superior competence in color discrimination, according to ISO/TR 28642:2016. All evaluators performed the Ishihara color-blindness test. The evaluations were carried out in a room with walls and floors in light colors, under natural lighting that corresponds to “midday light,” and next to the window, using visualization geometry of 0°/45°. With the teeth on a neutral gray background, the observers performed blind visual assessments of all teeth in a random order. The evaluators observed the teeth from 25 cm away and had 25 s to classify each specimen. After each tooth was evaluated, the observers were allowed to look at a neutral blue background to avoid eyestrain.

To determine the color match between the composites used and the teeth, the visual color-match score values were expressed numerically as follows, where 1 is the mismatch/completely unacceptable, 2 is the poor match/hardly acceptable, 3 is the good/acceptable match, 4 is the close match/small difference, and 5 is the exact match/no color difference; this scale is in accordance with ISO/TR 28642:2016. The results were recorded, and mean values were calculated.

Descriptive measurements related to the difference in color obtained through instrumental means were analyzed using the mean and standard deviation, wherein the means were compared using ANOVA, applying the Bonferroni post hoc test. In the analysis of visual color correspondence, the frequency distributions of the correspondence classifications for each evaluator, stratified by comparison groups, were presented. The significance adopted in the analysis was 5% (*p* < 0.05); the software used was STATA version 14, and GraphPad Prism version 6.0 was used to make the graphs.

## 3. Results

### 3.1. Instrumental Assessment

**Figure 1:**
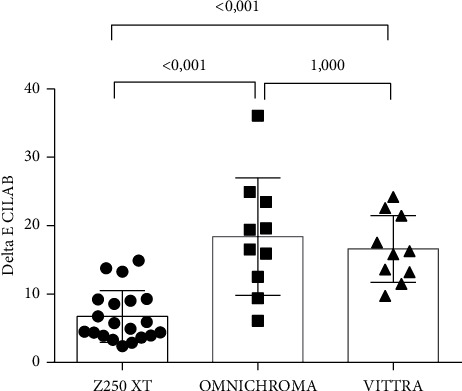
Graph illustrating color-matching measurements according to CIELAB.

Comparing the color difference values (ΔE) of the composite resins tested in the present study using CIELAB color coordinates, it was observed that there was a statistically significant difference between the groups (G1, G2, and G3) (ANOVA, *p* < 0.001). Comparing the groups two by two, using the Bonferroni post hoc test, there was a statistically significant difference between Z250 XT (G1) and OC (G2) (*p* < 0.001) and between Z250 XT and Vittra (G3) (*p* < 0.001), but there was no significant difference between the OC and Vittra groups (*p*=1.000) (Figure 1).  Table 2 presents the mean values of ΔE CIELAB and the standard deviations of the groups.

**Figure 2:**
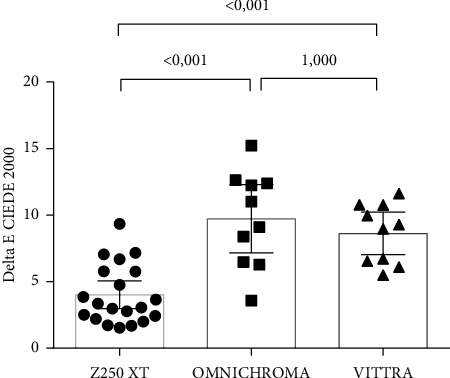
Graph illustrating color-matching measurements using formula CIEDE2000.

According to the CIEDE 2000 formula, there was a statistically significant difference between the groups (G1, G2, and G3) (ANOVA, *p* < 0.001). Comparing the groups two by two, using the Bonferroni post hoc test, there was a statistically significant difference between the composite resin Z250 XT (G1) and OC (G2) (*p* < 0.001), as well as between the composite resin Z250 XT and the Vittra (G3) (*p* < 0.001), but there was no significant difference between the OC and Vittra groups (*p*=1.000) (Figure 2).  Table 3 presents the mean values of ΔE using the CIEDE 2000 formula and the standard deviations of the groups.

### 3.2. Visual Assessment

For visual assessment, regardless of the assessment group, 77.49% of the teeth were within the acceptable color match classification, with the highest value being within the close match/small difference scale (38.33%). In the control group (G1), which was restored with Z250 XT composite resin, 66.67% of the teeth were classified as having an acceptable color match. The teeth of the test groups (G2 and G3), which were restored with single-tone resins, also showed a visual classification of color matching within acceptable limits, with values of 76.67% for OC and 80.00% for Vittra. The values obtained in each range of the visual assessment scale per evaluator for each group are shown in Table 4.

## 4. Discussion

Color matching is associated with the acceptability of restoration by both professionals and patients. The development of composite resins with a reduced number of colors, which simplifies the color selection process without impairing the esthetic result of the restorative procedure, represents an excellent advancement in the field of dental materials [9]. The null hypothesis of the present study was rejected because statistically significant differences were observed in the total color differences between the composite resins tested (*p* < 0.001). The intraoral spectrophotometer calculated the *L*^*∗*^, *a*^*∗*^, and *b*^*∗*^ values for each tooth specimen before and after restoration, resulting in the ΔΕ values.

To obtain a better correlation with visual perception, the ISO and CIE currently recommend using the CIEDE 2000 color difference formula (ISO/CIE 11664-6:2014). In our study, the total color difference (ΔΕ) was evaluated for AT and PT for both CIEDE 2000 and CIELAB. The 50% : 50% PT threshold for CIELAB was defined as 1.2 (ΔΕ < 1.2); such a limit indicates that 50% of the observers are able to detect the differences, whereas 50% are not. A shade difference below 2.7 was defined as the limit of AT for composite resin restorations. However, a color difference greater than 2.7 may still be clinically acceptable [20].

Considering the CIEDE 2000 formula, the values were also evaluated in terms of 50% : 50% of PT and 50% : 50% of AT, wherein the thresholds were determined as 0.8 and 1.8, respectively, as reported in previous studies [23, 24]. Several studies reported a better fit of the CIEDE 2000 formula in the assessment of visual parameters (95% agreement with visual findings, as opposed to 75% for the CIELAB formula, thus supporting its use in tooth color research) [7, 20].

In our results, ΔΕ was above the PT and AT thresholds for both CIELAB and CIEDE 2000. None of the materials presented a ΔΕ 00 value below 1.8, which represents the threshold value for the AT level, whereas 0.8 is the threshold of restoration PT. These results are in line with those of previous studies in which the evaluated composite resins obtained values above the AT and PT thresholds [7, 12, 27]. Furthermore, a numerically small ∆E value does not necessarily correspond to the best color match because of uneven eye sensitivity in regard to differences in hue, value, and chroma [7].

The Z250 XT multishade resin showed a statistically significant difference in relation to the single-shade resins in both ΔE ab and ΔE 00 evaluations. The average value of ΔE 00 for the multishade resin was 4.06; therefore, it was closer to the level of AT compared to the single-shade resins OC (9.83) and Vittra (8.72). This result is in line with other studies in which the multishade resin also showed better color-matching values (lower ΔE) [2, 27]. In a study by Kobayashi et al. [14], the tested multishade composite resins were used only in the A2 shade to restore teeth with various shades of color, which may explain the higher values of ΔE presented for these resins in comparison with the single-shade resin OC, considering that only one shade of color from the multishade resin may not have been sufficient to mimic the colors of other shades [14].

There was no statistically significant difference between the single-shade resins evaluated in the present study; however, Vittra APS Unique showed lower values of ΔΕ ab and ΔΕ 00 compared to OC. In a recently published study [4] that evaluated the color-matching potential of four single-tone resins on extracted human incisors, the ∆E 00 values were also lower for Vittra APS Unique resin (4.75) than for OC (5.43). However, in this study, a comparison with multishade composite resin was not performed. Color matching may vary depending on the shade [28]. Studies indicate a better matching of single-shade resins with lighter color tones [7, 12, 29].

Using instruments to assess color matching has the advantage of reducing imperfections and inconsistencies that are presented in visual matching, as discussed previously [30]. The equipment used for instrumental evaluation in this study was a VITA Easyshade Advance 4.0 spectrophotometer. This spectrophotometer was also used in several other studies that evaluated the color matching of composite resins [7, 13, 31, 32].

Another factor that can interfere with the color-matching values of composite resins is the size of the restored cavity. The “chameleon effect” in composite resins is influenced by the restoration size. Paravina et al. [33] concluded that color matching for restorations increases with decreasing cavity size and increasing translucency of the filling material. Altınışık and Özyurt [4] obtained lower values of ∆E 00 for single-shade composite resins in their findings compared to those presented in the current study, in which standardized cavities with a depth of 2 mm and a diameter of 7 mm were created, which may have contributed to the lowest values of ∆E 00 in these materials.

Color is one of the most important esthetic parameters in dentistry. Several factors can affect the color of composite resins, such as color properties (lightness, chroma, and hue) and translucency [4]. The layers of natural teeth have irregular morphologies with irregular surface structures [34]. All these factors contribute to the difficulty of proper selection and color matching of composite resin restorations.

Furthermore, there are some limitations to measuring tooth color using only instrumental devices. Human teeth are small and curved, which can lead to poor color readings because a considerable part of the light that strikes the tooth surface is lost. This represents one of the disadvantages of equipment such as dental spectrophotometers. Therefore, these devices are recommended as aids in the visual assessment of color matching but should not replace it [30].

Visual assessment is the most commonly used method for assessing color in clinical dental practice [23]. A combination of visual and instrumental techniques has been recommended for better PT and AT of the color assessment of composites [35]. Therefore, it is recommended that the instrumental determination of color is always accompanied by experienced human visual perception [30].

In the present study, visual evaluation was performed by three evaluators with superior competence in the field of esthetic dentistry (graduate students, doctoral students, and specialists in restorative dentistry). All the composite resins tested in the present study showed color-matching values within the range of acceptable values (good/acceptable, close/small, or exact difference/no color difference). However, the single-shade resins showed better visual color matching than the multishade composite resins. Vittra APS Unique resin showed matching values of color within the range of even higher acceptable values (80.00%) when compared to OC (76.67%), which is in agreement with the results presented in the instrumental evaluation (ΔE ab and ΔE 00). The visual method is subjective, but the visual judgment of color matching or mismatching is often the deciding factor in overall patient acceptance [36].

The OC single-shade composite resin used in the experimental group in our research was also tested in several other studies, showing both acceptable [5, 9–12, 14, 16] and unacceptable color-matching results [2, 7, 13]. Color difference evaluation parameters other than the AT and PT thresholds used in this study are found in these studies, which may also explain the differences in the results presented. The color adjustment potential (CAP) was introduced by Paravina et al. [37] and represents another parameter used to verify the mixing effect of composite resins and has been used as an evaluation method in several studies [10, 14]. CAP is a parameter that aims to describe and quantify the interaction of two components: perceptual, which is evaluated visually (CAP-V), and physical, which is evaluated using a color measurement instrument (CAP-I) [36]. The physical component of the mixture results from translucency and can be quantified as the ratio of the color difference values between two objects under two conditions: one surrounded by the other and separately [37].

According to the manufacturer of OC resin, this observed “chameleon effect” can be achieved because of the inclusion of uniformly sized 260 nm spherical fillers, which can generate a red to yellow color as ambient light passes through it [2]. OC resin was developed with intelligent color technology and uses the concept of structural color, wherein the material itself weakens or amplifies specific wavelengths of light to blend with tooth color, unlike other composite resin systems that add red and yellow pigments to color the material [7].

The filler particles present in the composite resin can influence the light transmission characteristics of the material [38]. Furthermore, the blending effect of restoration can be influenced by the scattering and diffuse refraction of light through the composite resin [9]. The incident light is reflected by the filler, organic resin matrix, pigments, and background color in the composite resin, resulting in perception as a certain color [39].

Nanoparticulate composites seem to exhibit better polishing and greater final brightness of the restoration, with a consequent reduction in surface roughness compared to conventional hybrid composites [14]. This may also lead to better color matching, as indicated by Chen et al. [9], where the supra-nano-filled composite showed better color-matching capability with all cavity shade class I composites simulated with composite resin in silicone molds compared to composite resin with microhybrid filler particles. Furthermore, composite resins containing supra-nanospheric filler particles with a uniform size of 260 nm in diameter demonstrated better color matching than composite resin containing supra-nanospheric charges with a diameter of 150 nm [40].

Composites containing Bis-GMA are more translucent than those without; thus, the organic matrix of the resin affects the translucency of the material. Furthermore, in some studies, translucency was positively correlated with the material color-matching effect [5, 7]. OC and Vittra APS Unique do not have Bis-GMA in their resinous organic matrix (as presented in their technical reports), which can reduce the translucency of these materials and consequently their “chameleon effect.”

As the inherent optical properties of materials are closely associated with their chemical and physical properties, variation in the composition of composite resins makes it difficult to generalize the associations between ingredients and perceived color [1]. Regardless of the technology used by manufacturers to achieve better color matching, their use would greatly simplify the clinical color matching of restorations and reduce turnaround time [9].

The present study has limitations because it was an in vitro study in which extracted human teeth were used. The results presented in this study may be influenced by several factors associated with both tooth structure and the evaluated composite resin. Variables such as the evaluation time, cavity type, cavity depth, evaluation methods (instrumental and/or visual), brand of single-shade composite resin tested, color of material, type of specimen/sampling unit evaluated, and even brand commercial use of the composite resin used in the control group may influence studies of this type. The sizes of the cavities made in this study were the same for all restorations; however, it may be interesting to conduct a similar study, wherein the cavity sizes of human teeth are varied. In addition, the properties of single-shade composite resins, such as color stability in human teeth and translucency, among other optical properties, should also be evaluated in future studies. Clinical studies must be performed to confirm the results of this in vitro study.

## 5. Conclusion

In the evaluation using the spectrophotometer, the multishade resin presented lower values of ΔE for both CIELAB and CIEDE 2000, that is, better color correspondence than the single-shade resins. In the visual evaluation, all groups demonstrated acceptable color matching; however, single-shade composite resins showed better matching values than the multishade resin.

## Figures and Tables

**Table 1:** Materials used in the study.

Materials	Composition
Omnichroma (Tokuyama, Tokyo, Japan)	Inorganic filler particles: 79% by weight (68% by volume) of spherical zirconium silica filler (average particle size 0.3 *µ*m, range 0.2–0.4 *µ*m). Organic matrix: UDMA, TEGDMA, mequinol, dibutilhidroxitolueno e UV absorber
Vittra APS Unique (FGM, Joinville, Brazil)	Organic matrix: UDMA, TEGDMA. Inorganic filler particles: active ingredients: methacrylate monomer blend, photoinitiator composition (APS), co-initiators, stabilizers, and silane. Inactive ingredients: boron-aluminum-silicate glass
Filtek Z250 XT (3M ESPE, St. Paul, MN)	Organic matrix: Bis-GMA, UDMA, Bis-EMA, PEGDMA e TEGDMA, fluorescent agents, pigments, stabilizers, and initiators. Inorganic filler particles: zirconia/silica: 3 *µ*m or less, zirconia/silica cluster, surface-treated silica: 20 nm, 67.8% by volume

Bis-EMA, bisphenol A ethoxylated dimethacrylate; Bis-GMA, bisphenol A-glycidyl methacrylate; TEGDMA, triethylene glycol dimethacrylate; UDMA, urethane dimethacrylate.

**Table 2:** Comparison of color difference measures—Delta E CIELAB between groups.

Groups	*n*	Mean ± SD	*p* value, G1 × G2	*p* value, G1 × G3	*p* value, G2 × G3
Z250 XT (G1)	20	6.80 ± 3.8	<0.001	<0.001	1.000
Omnichroma (G2)	10	18.6 ± 8.6			
Vittra APS Unique (G3)	10	16.8 ± 4.9			

ANOVA test: *p* value <0.001.

**Table 3:** Comparison of color difference measurements using Delta E CIEDE2000.

Groups	*n*	Mean ± SD	*p* value G1 × G2	*p* value G1 × G3	*p* value G2 × G3
Z250 XT (G1)	20	4.06 ± 2.3	<0.001	<0.001	1.000
Omnichroma (G2)	10	9.83 ± 3.6			
Vittra APS Unique (G3)	10	8.72 ± 2.3			

ANOVA test: *p* value <0.001.

**Table 4:** Visual assessment of color matching between the 3 independent raters stratified by groups.

Color matching	Evaluator 1 number (%)	Evaluator 2 number (%)	Evaluator 3 number (%)	Total (%)
*Independent of the group*				
Exact match/no difference in color	2 (5.0%)	11 (27.5%)	0 (0%)	10.83
Very good match/small difference	17 (42.5%)	15 (37.5%)	14 (35.0%)	38.33
Good match/acceptable	9 (22.5%)	8 (20.0%)	17 (42.5%)	28.33
Poor match/hardly acceptable	9 (22.5%)	6 (15.0%)	9 (22.5%)	20.00
Mismatch/totally unacceptable	3 (7.5%)	0 (0%)	0 (0%)	2.50

*Z250 XT (G1)*				
Exact match/no difference in color	0 (0%)	9 (45%)	0 (0%)	15.00
Very good match/small difference	6 (30%)	4 (20%)	5 (25%)	25.00
Good match/acceptable	5 (25%)	2 (10%)	9 (45%)	26.67
Poor match/hardly acceptable	7 (35%)	5 (25%)	6 (30%)	30.00
Mismatch/totally unacceptable	2 (10%)	0 (0%)	0 (0%)	3.33

*Omnichroma (G2)*				
Exact match/no difference in color	0 (0%)	0 (0%)	0 (0%)	0.00
Very good match/small difference	5 (50%)	4 (40%)	2 (20%)	36.67
Good match/acceptable	2 (20%)	5 (50%)	5 (50%)	40.00
Poor match/hardly acceptable	2 (20%)	1 (10%)	3 (30%)	20.00
Mismatch/totally unacceptable	1 (10%)	0 (0%)	0 (0%)	3.33

*Vittra APS Unique (G3)*				
Exact match/no difference in color	2 (20%)	2 (20%)	0 (0%)	13.33
Very good match/small difference	6 (60%)	7 (70%)	7 (70%)	66.67
Good match/acceptable	2 (20%)	1 (10%)	3 (30%)	20.00
Poor match/hardly acceptable	0 (0%)	0 (0%)	0 (0%)	0.00
Mismatch/totally unacceptable	0 (0%)	0 (0%)	0 (0%)	0.00

## Data Availability

The data used to support this study are available from the corresponding author upon request.
